# Platinum-based chemotherapy in triple-negative breast cancer: A meta-analysis

**DOI:** 10.3892/ol.2012.1093

**Published:** 2012-12-28

**Authors:** MIAO LIU, QIN-GUO MO, CHANG-YUAN WEI, QING-HONG QIN, ZHEN HUANG, JIE HE

**Affiliations:** Breast Surgery Department of Tumor Hospital, Guangxi Medical University, Nanning, Gaungxi 530021, P.R. China

**Keywords:** meta-analysis, triple-negative breast cancer, platinum

## Abstract

Triple-negative breast cancer (TNBC) tumors do not express estrogen, progesterone or HER2/neu-receptors. There are no specific treatment guidelines for TNBC patients, however, it has been postulated that their phenotypic and molecular similarity to BRCA1-associated cancers would confer sensitivity to certain cytotoxic agents, including platinum. The aim of this meta-analysis was to evaluate the clinical outcome of breast cancer patients treated with platinum-based chemotherapy who had TNBC compared with those with non-TNBC. Electronic (MEDLINE, EMBASE and Cochrane Library databases) and manual searches were conducted throughout December 2011 to identify trials evaluating the use of platinum-based chemotherapy for patients with breast cancer. The methodological quality was assessed in accordance with the QUOROM statement. Seven studies met the eligibility criteria, with a total of 717 patients. Of these patients, 225 were TNBC patients (31%), 492 were non-TNBC patients (69%), 275 received platinum-based neo-adjuvant chemotherapy and 442 had advanced/metastatic breast cancers. The results showed that during neo-adjuvant chemotherapy, the clinical complete response (cCR) rate and the pathological complete response (pCR) rates were significantly higher for the TNBC group compared with the non-TNBC group (OR, 2.68; 95% CI, 1.69–6.57; P=0.03 and OR, 2.89; 95% CI, 1.28, 6.53; P= 0.01, respectively). However, in advanced/metastatic breast cancers, the cCR, partial response (PR) and the disease control rates for the TNBC group were not significantly different compared with the non-TNBC group. The 6-month progression-free survival (PFS) rate for the TNBC group was higher than that of the non-TNBC group in all patients (OR, 1.81; 95% CI, 1.11–2.96; P= 0.02). However, the 1- and 2-year PFS rates were not significantly different (OR, 1.42; 95% CI, 0.69–2.92; P=0.35 and OR, 1.11; 95% CI, 0.35–3.52; P= 0.85, respectively). Furthermore, the PFS rates were not significantly different between the groups in patients with advanced/metastatic breast cancer. In conclusion, platinum-based chemotherapy in the breast cancer patients with TNBC showed an improved short-term efficacy compared with the non-TNBC group during neo-adjuvant chemotherapy, but has not yet been demonstrated to have an improved effect in advanced breast cancer.

## Introduction

Triple-negative breast cancer (TNBC) was first identified by Perou and Sorlie of Stanford University and was defined as tumors that do not express estrogen receptor (ER), progesterone receptor (PR) and HER2. Of all breast cancers, 12–20% are TNBC (1). The majority of these tumors are high-grade or poorly differentiated tumors and treatment options for TNBC have been limited by the lack of targeted therapies, so the prognosis is poorer than for other types of breast cancer. It has been postulated that their phenotypic and molecular similarity to BRCA1-associated breast cancers may prove useful in terms of treatment (2). The DNA of normal cells may be damaged in a number ways which activate regulation by the DNA repair-associated protein, BRCA1. When BRCA1 mutations occur, the DNA repair function is not regulated and there is an inherited correlation between the BRCA1 gene and the pathogenesis of breast cancer (3). According to previous studies, ∼70% of breast cancer cases exhibit the correlation between the BRCA1 gene immune group and TNBC (4). Platinum is a common second-line antitumor drug in breast cancer chemotherapy. It has been suggested that platinum may be an effective drug treatment for breast cancer with genetic mutations in the BRCA1 gene. An *in vitro* study concerning the BRCA1 mutation in rat breast epithelial cells showed that platinum and gemcitabine exhibited superior outcomes compared with the first-line chemotherapy drugs anthracycline, paclitaxel and fluorouracil (5). Platinum drugs for TNBC may also have improved curative effects. A study by Sirohi *et al*(6) reported that when 28 patients with TNBC were treated with 4 cycles of cisplatin as the foundation of neo-adjuvant chemotherapy (75 mg/m^2^, day 21), 6 patients (21%) achieved pathological complete response (pCR) and 18 (64%) achieved clinical complete response (cCR) or partial response (PR). Another study used cisplatin, epirubicin and docetaxel in a single solution administered weekly. Of the 74 TNBC patients, 46 achieved pCR and the total five-year disease-free survival (DFS) rate was 76% (7). Although numerous clinical studies have demonstrated the superior efficacy of platinum treatments for TNBC, the simple clinical efficacy has been observed mainly in small numbers of TNBC patients and non-TNBC controls, with each sample size being small and of variable quality. It is therefore necessary to use a systematic evidence-based system to evaluate the effect of platinum-based chemotherapy in treating TNBC and the long-term survival using previous studies to provide higher quality clinical evidence.

## Materials and methods

### 

#### Identification of trials

The MEDLINE, EMBASE and Cochrane Library databases were systematically searched until December 2011. Comparative studies were identified using any of the following keywords: cisplatin, platinum, carboplatin, paraplatin, oxalipatin, lobaplatin, breast cancer and breast carcinoma. The search was not limited to controlled or randomized trials to minimize the chance of missing a study. A manual search of the relevant references was performed to identify further relevant trials. There were no date or language restrictions. Studies involving neo-adjuvant therapy for advanced/metastatic cancers were excluded. The studies identified through the search were independently screened by two authors (M.L. and Q-G.M.) for inclusion. Any disagreements were arbitrated by a third author (CY.W.).

#### Outcome measures

The primary outcomes evaluated in the present review were the complete response (CR), PR, pCR, clinical benefit, DFS, progression-free survival (PFS) and overall survival (OS) rates. The secondary outcome was the adverse effects of treatment/toxicity (including withdrawals and discontinuations).

#### Quality assessment

The present systematic review was conducted in accordance with the Quality of Reporting of Meta-analyses (QUOROM) statement (8). Two authors independently evaluated all included trials based on randomized sequence generation, allocation concealment, blinding of outcome assessors and reporting of an intention-to-treat analysis. Trials were considered to be of low quality if they reported none of the items, medium quality if they reported on <3 and of high quality if they reported on 3 or 4.

#### Data extraction

Two authors independently extracted the data concerning the author details, year, methodological quality, number of patients, patient characteristics, interventions (i.e., drugs, schedule and number of therapeutic sessions) and outcomes using a data extraction form. Discrepancies were resolved by consensus. When multiple publications of the same trial were identified, data were extracted and reported as a single trial.

#### Statistical analysis

The Cochrane Collaboration Review Manager 4.2.2 statistical software was used for for meta-analysis. To test for heterogeneity in the included studies and analyze the statistical heterogeneity using the χ^2^ test, the significance level was set at P≤0.10. When heterogeneity existed between the results, I^2^ heterogeneity quantitative analysis was used and the significance level set at 50%, so I^2^>50% indicated heterogeneity in the results. If the test results indicated that the heterogeneity between the groups was not signficant, then a fixed-effects model was used for meta-analysis. On the contrary, if I^2^> 50%, then there was heterogeneity. After been processed the heterogeneity still can not be eliminated, and is intended merger analysis using a random-effects model. A random-effects model was used for pooled analysis if there was clinical heterogeneity in a subgroup analysis and treatment heterogeneity was not eliminated. The standardized mean difference (SMD) or weighted standard deviation (MD) were the effect indicators of the present study and the 95% CI was used for the efficacy analysis statistics.

## Results

### 

#### Description of studies

**Figure 1:**
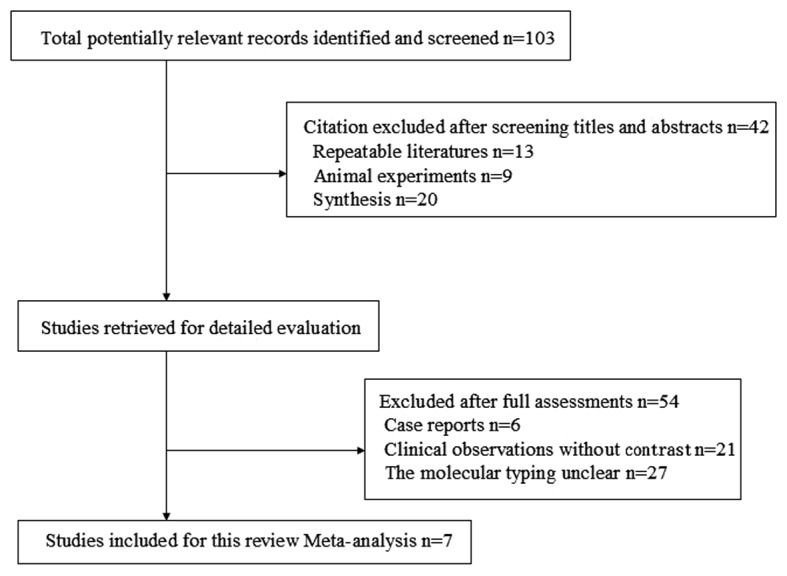
Flow chart of studies identified, included and excluded.

A total of 103 references were identified and 42 studies were excluded by reading the titles and abstracts to identify the repeated studies, animal experiments and synthesis. The studies were read further to identify case reports, clinical observations without contrast and studies where the molecular typing was unclear, to exculde a total of 54 studies. Ultimately, seven studies were selected with a total of 717 patients. The reference flow is shown in Fig. 1.

#### Quality of included studies

The seven studies were retrospective cohorts, including 108 patients (9–14). The basic characteristics are shown in Table I.

The only platinum drugs used in the studies were cisplatin and carboplatin. The study by Sirohi *et al*(9) analyzed neo-adjuvant chemotherapy in 94 and 155 cases of advanced/metastatic breast cancer, so the study was divided into Sirohi 1 *et al* and Sirohi 2 *et al*, neo-adjuvant and advanced/metastatic cases, respectively, to analyze the outcome, as shown in Tables II, III and IV. Two studies (10,11) were neo-adjuvant chemotherapy studies and the remaning four (12–15) were of advanced/metastatic breast cancer. Of the seven studies of TNBC, five (9–12,14) had a clear description of ER and PR detection using an immunohistochemical method, where ER and PR were defined as negative in TNBC. HER2 testing was performed differently and the negative standard was different. One study (9) described an immunohistochemical assay for HER2. HER2 immunohistochemistry or FISH gene amplification were used to define negative HER2 expression. The neo-adjuvant chemotherapy, intervention and outcome measures used in the studies are shown in Table II and another study of a platinum single-agent neo-adjuvant chemotherapy used in an observational study of clinical efficacy was added as a comparison (6). The studies were divided into groups of three; except the Sirohi 1 study (9) which used doxorubicin, the other 2 studies (10,11) used a drug combination of carboplatin and paclitaxel. The Sirohi 1 (9) study reports only the cCR as certain cases did not receive surgical treatment; the other 2 studies both have pCR data. Sirohi 1 (9) reported that in certain cases the cisplatin was replaced with carboplatin (AUC=5), due to adverse reactions causing renal toxicity, neutropenia and anemia.

#### Neo-adjuvant cCR rate

**Figure 2:**
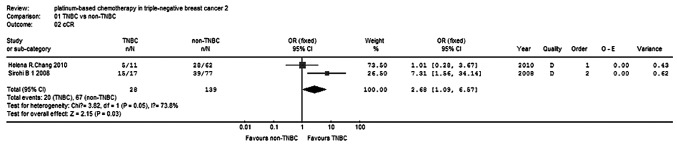
Clinical complete response rate: neo-adjuvant.

Two studies (9,10) reported the cCR rate for 167 patients and the test for heterogeneity was statistically significant (P=0.05, I^2^=73.8%), indicating the presence of a large statistical heterogeneity, so a random-effects model was used for the pooled analyses. The meta-analysis results showed that the difference in the cCR rate was statistically significant (OR, 2.68; 95% CI, 1.69–6.57; P= 0.03; Fig. 2) between the the TNBC group and non-TNBC group with regard to neo-adjuvant therapy. The cCR rate of the TNBC group was 2.68-fold higher than that of the non-TNBC group.

#### pCR rate

**Figure 3:**
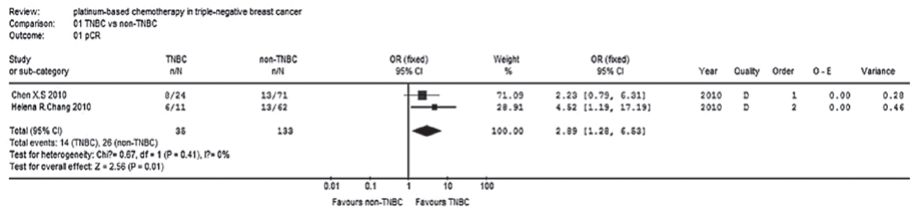
Pathological complete response rate: neo-adjuvant.

Two studies (10,11) reported the pCR for 168 patients and the test for heterogeneity was not statistically significant (P=0.41, I^2^=0%), so the fixed-effects model was used. The meta-analysis results (Fig. 3) showed that the difference in pCR rates was statistically significant (OR, 2.89; 95% CI, 1.28–6.53; P=0.01) between the TNBC and non-TNBC groups, with regard to carboplatin-paclitaxel chemotherapy. The effect of the neo-adjuvant therapy on the TNBC group was superior to that of the non-TNBC group.

#### OS and DFS rates

Only one study (9) reported the OS and DFS rates for neo-adjuvant platinum-based chemotherapy which included 94 cases with descriptive analysis. The 5-year OS rates in the TNBC and the non-TNBC groups were 0.65 and 0.8, respectively, while the 10-year OS rates in the TNBC and the non-TNBC groups were 0.53 and 0.65 respectively. The median DFS times were 68 and 90 months, respectively, with no significant difference observed between the two groups (P= 0.6), while the median OS times were 125 and 169 months, respectively. The distant recurrence and survival of the patients treated using neo-adjuvant chemotherapy with epirubicin, cisplatin and 5-Fu was not significantly different between the TNBC and the non-TNBC groups.

### Advanced/metastatic disease

#### Response

**Figure 4:**
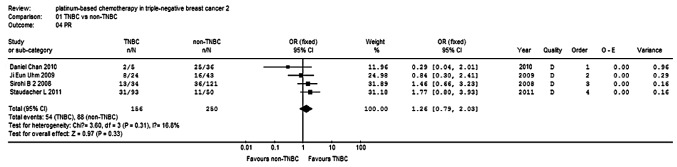
Partial response rate: advanced/metastatic.

**Figure 5:**
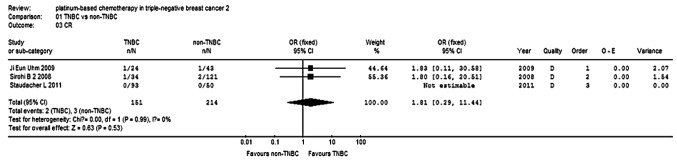
Clinical complete response rate: advanced/metastatic.

**Figure 6:**
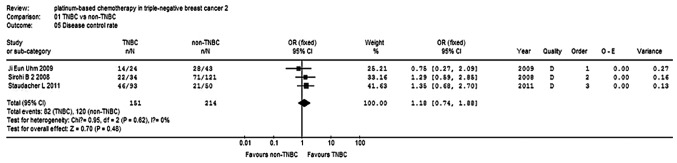
Disease control rate (CR+PR+SD): advanced/metastatic. CR, complete response; PR, partial response, SD, stable disease.

Four studies (9,13–15) reported PR in 406 patients, and three studies (9,14,15) reported cCR in 365 patients. There was no statistical heterogeneity within each sub-group, so a fixed-effects model was used to analyze the combinations. The results show that the PR and cCR of the TNBC group were 1.26- and 1.81-fold that of the non-TNBC group, respectively, but no significant differences were observed (P=0.33, P=0.53; Figs. 4 and 5). The clinical benefit rate (cCR + PR + stable disease) of the TNBC group was 1.18-fold higher than that of the non-TNBC group, but no significant differences were observed (P=0.48; Fig. 6).

#### OS and PFS rates

**Figure 7:**
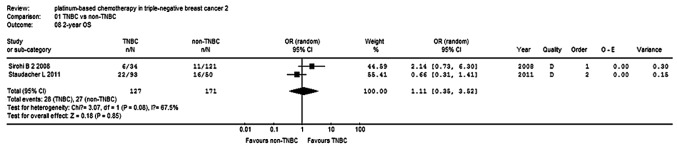
2-year overall survival rate: advanced/metastatic.

**Figure 8:**
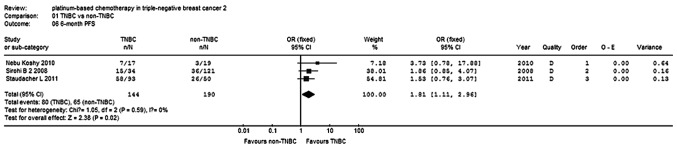
6-month progression-free survival rate: advanced/metastatic.

**Figure 9:**
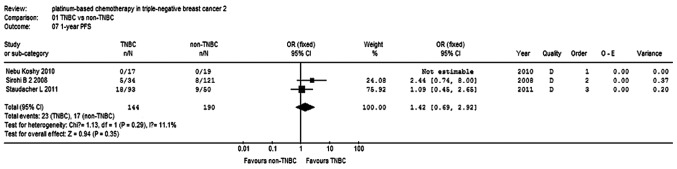
1-year progression-free survival rate: advanced/metastatic.

Three studies (9,12,15) reported 6-month and 1-year PFS rates for a total of 234 patients, while two studies reported 2-year OS rates for 298 patients. There was no statistical heterogeneity within the subgroups, so a fixed-effects model was used to analyze the combinations (Fig. 7). The 6-month PFS rate of the TNBC group was higher compared with the non-TNBC group (OR, 1.81; 95% CI, 1.11–2.96; P=0.02; Fig. 8). The 1-year PFS rate was not significantly different between the two groups (OR, 1.42, 95% CI, 0.69–2.92; P=0.35; Fig. 9).

## Discussion

TNBC is a high-risk breast cancer due to the younger age of patients, poorly differentiated tumors and shortened survival that lacks the benefit of targeted therapies. Platinum-based chemotherapy in TNBC is a popular research topic, although there are significant differences between the various studies concerning the drug use, dose, cycle, patient ages, tumor stages and patient physical condition, so the results of the studies are not the same. The results of the present study show that, for cases receiving neo-adjuvant or adjuvant chemotherapy with platinum, the pCR and cCR rates of the TNBC group were significantly higher than those of the non-TNBC group. However the long-term recurrence and survival exhibited little difference between the groups. Sirohi *et al*(9) reported a 5-year OS rate of 65% in TNBC patients treated with neo-adjuvant/adjuvant chemotherapy, while Silver *et al*(6) reported that with the single-agent cisplatin, six (21%) of the 28 patients achieved pCR and 18 (64%) achieved cCR or PR. However, the 5-year OS rate in non-TNBC patients has been reported to be 85% higher than that of TNBC patients (9). According to two studies (16,17), the TNBC and non-TNBC patients who achieved pCR had similar OS rates. Of the neo-adjuvant chemotherapy patients achieving pCR and the non-pCR cases, the 2-year recurrence-free rates were 93.8 and 78.4%, respectively, while the 3-year recurrence-free rates were 83.3 and 58%, respectively. Therefore, for neo-adjuvant chemotherapy used to treat TNBC patients, pCR is a significant indicator of a good prognosis.

In addition, for the platinum treatment in the metastatic TNBC and non-TNBC groups, the overall response (OR) rates were similar and the long-term OS and DFS were no significantly different. It is noteworthy that with the platinum treatment, the TNBC group often had longer PFS and chemo-therapy survival times (Table V). Koshy *et al*(12) noted that, compared with non-TNBC patients, the disease progression risk of TNBC patients was reduced by 47%. However, previous studies appear to show higher long-term recurrence rates in the TNBC group with shorter DFS and lower OS. The present study suggests that, overall, the two groups were not significantly different and it may be proposed that platinum treatment in the TNBC group patients with prolonged survival times and improved disease progression times was superior to that of the non-TNBC group.

The present analysis included seven retrospective cohort studies of varying quality and had the following limitations. Since the observation time was long, there may be bias as many cases were lost in follow-up. Study exposure factors that affect the drug treatment, dose and cycle, including cases where non-TNBC patients receive endocrine therapy and targeted therapy, which the TNBC cases lack, cause a large bias. There were certain differences between the studies with regard to the source of subjects, disease classification, age, illness, physical fitness and primary or secondary baseline information, which lead to larger clinical heterogeneity. A lack of detailed data on the DFS and PFS period data prevented the analysis of count data, such as the OS and PFS times.

In summary, the efficacy of platinum in TNBC treatment was demonstrated in the short- and long-term, subject to further research and feasibility studies.

The present systematic review included studies where the overall quality was not high and it requires more rigorous design of high-quality randomized controlled studies to reduce and remove bias that may exist in the study results. Future research should focus more on comparisons of the therapeutic effects of platinum compared with other chemotherapy drugs, in order to allow more valuable quality of life studies of TNBC patients.

## Figures and Tables

**Table I:** Baseline characteristics of randomized controlled trials included the meta-analysis.

Authors (Ref.)	Year	Country	Clinical stage	Chemotherapy
Sirohi, *et al* 1 (9)	2008	UK	T2–T4, N0–N3, M0	EPI + DDP + 5-Fu
Chang, *et al*(10)	2010	US	T2–T4, N0–N3, M0	Carboplatin + Doc
Chen, *et al*(11)	2010	China	II–IIIC	Paclitaxel + Carboplatin
Koshy, *et al*(12)	2010	US	Metastatic	Carboplatin + Gemcitabine
Sirohi, *et al* 2 (9)	2008	UK	Advanced/metastatic	DDP or Carboplatin
Chan, *et al*(13)	2010	Singapore	Metastatic	Carboplatin + Gemcitabine
Uhm, *et al*(14)	2009	Korea	Advanced/metastatic	DDP or Carboplatin
Staudacher, *et al*(15)	2011	France	Advanced/metastatic	DDP or Carboplatin

EPI, epirubicin; DDP, cisplatin; 5-Fu, 5-fluorouracil; Doc, docetaxel.

**Table II:** Study characteristics for patients receiving chemotherapy for neo-adjuvant cancer.

Authors (Ref.)	Sample	Mean age (year)	Clinical stage	Mean tumor size (cm)	Drugs and dose	Surgery rate	Outcomes	Toxicities of grade III–IV
Sirohi, *et al* 1 (9)	TN: 17 Non-TN: 77	TN: 50 Non-TN: 46	T3–T4 Nx; M0	NR	5 FU: 200 mg/m^2^ DDP: 60 mg/m^2^ EPI: 60 mg/m^2^ day 1 for 21 days^*^6	6 (35%) P=0.005 54 (70%) P=0.007	cCR, PR, pD	NR
Chang, *et al*(10)	TN: 11 Non-TN: 62	49.6	T2–T4 Nx; M0	7.75	DOC: 75 mg/m^2^ Carboplatin: AUC=6 day 1 for 21 days^*^4	71	pCR, cCR, 2-year OS, 5-year OS	Neutropenia, leukopenia, lymphopenia, febrileneutropenia
Chen, *et al*(11)	TN: 24 Non-TN: 84	51	II–IIIC	NR	Carboplatin: AUC=2 Taxol: 180 mg/m^2^ days 1+8+15 for 28 days^*^4	108	pCR	Neutropenia, thrombocytopenia, anemia, skin toxicity
Silver, *et al*(6)	TN: 28	29–69	II–III	3.7 (2.0–7.0)	DDP: 75 mg/m^2^ day 1 for 21 days^*^4	28	pCR: 6 (21%) cCR+PR: 18 (64%) PD: 4 (14%)	Tinnitus, neutropenia, hyperkalemia, elevation of ALT/AST, nausea, myalgia, skin toxicity

TN, triple-negative breast cancer; Doc, docetaxel; DDP, cisplatin; cCR, clinical complete response; pCR, pathological complete response; PD, progressive disease; OS, overall survival; PFS, progression - free survival; TTP, time to progression; Nx, status of lymph node metastasis is unclear; NR, not reported in the text.

**Table III:** Patient characteristics for those receiving chemotherapy for advanced/metastatic cancer.

Authors (Ref.)	Mean age (years)	Sample	Node invasion (%)	Visceral metastasis (%)	Bone metastasis (%)	Brain metastasis (%)	Drugs and dose
Sirohi, *et al* 2 (9)	TN: 47 Non-TN: 53	TN: 34 Non-TN: 121	TN: 9 (26) Non-TN: 9 (8)	TN: 21 (62) Non-TN: 101 (83)	TN: 4 (12) Non-TN: 11 (9)	NR	M: 6 or 8 mg/m^2^ days 1+8+22+36 V: 6 mg/m^2^ DDP: 50 mg/m^2^ (or carboplatin: AUC=5) day 1 for 42 days
Chan, *et al*(13)	52	TN: 5 Non-TN: 36	NR	NR	21 (51)	5 (12)	G: 1000 mg/m^2^ days 1+8 Carboplatin: AUC=5 day 1 for 21 days
Uhm, *et al*(14)	TN: 46 Non-TN: 44	TN: 24 Non-TN: 43	NR	NR	NR	NR	DDP or carboplatin
Staudacher, *et al*(15)	TN: 48.4 Non-TN: 51.5	TN: 93 Non-TN: 50	TN: 60 (64.5) Non-TN: 30 (60.0)	TN: 69 (74.2) Non-TN: 41 (82)	NR	TN: 18 (19.4) Non-TN: 7 (14.0)	DDP: 120 (83.9%) Carboplatin: 23 (16.1%)
Koshy, *et al*(12)	TN: 47.5 Non-TN: 50.2	TN: 17 Non-TN: 19	TN: 12 (71) Non-TN: 6 (32)	NR	TN: 11 (65) Non-TN: 9 (47)	TN: 7 (41) Non-TN: 4 (21)	DDP: 25 mg/m^2^ G: 1000 mg/m^2^ days 1+8 for 21 days (or days 1+8+15 for 28 days)
Liedtke, *et al*(16)	TN: 58	TN: 38	12 (32)	33 (87)	9 (24)	NR	DDP: 30 mg/m^2^ G: 750 mg/m^2^ days 1+8 for 21 days

TN, triple-negative breast cancer; M, mitomycin C; V, vinblastine; DDP, cisplatin; G, gemcitabine; NR, not reported in the text.

**Table IV:** Results and toxicities for patients receiving chemotherapy for metastatic/locally recurrent cancer.

Authors (Ref.)	Outcomes	Median number of courses (range)	Toxicities in grade III–IV
Sirohi, *et al*(9)	CR, PR, PD, PFS, OS	5 (1–8)	NR
Chan, *et al*(13)	PR, TTP	4 (1–6)	Leukopenia, neutropenia, anemia, thrombocytopenia, febrile-neutropenia, diarrhea, hyponatremia
Uhm, *et al*(14)	CR, PR, SD, PD	NR	NR
Staudacher, *et al*(15)	CR, PR, SD, PD, OS, PFS	4 (1–9)	Febrileneutropenia, neutropenia, thrombocytopenia, anemia
Koshy, *et al*(12)	PFS, OS^a^	3–5	NR
Liedtke, *et al*(16)	CR: 2 (5%) PR: 13 (35%) SD: 13 (35%) PD: 10 (27%) TTP	5	Leukopenia, febrileneutropenia, thrombocytopenia, anemia, alopecia, nausea/vomiting, asthenia

CR, complete response; PR, partial response; SD, stable disease; PD, progressive disease; OS, overall survival; PFS, progression-free survival; TTP, time to progression; NR, not reported in the text; OS^a^, overall survival after start of cisplatin-based chemotherapy.

**Table V:** Long-term effects of metastatic/locally recurrent cancer.

Study (Ref.)	PFS (months)	OS (months)	OS after start of PBCT	6 month PFS (%)	1 year PFS (%)	2 year OS (%)
Sirohi 2 *et al*(9)	TN: 6 Non-TN: 4 P=0.05	NR	TN: 11 Non-TN: 7	TN: 15 (44) Non-TN: 36 (30)	TN: 5 (14) Non-TN: 8 (7)	TN: 6 (17) Non-TN: 11 (9)
Uhm *et al*(14)	NR	TN: 21 Non-TN: 56	NR	NR	NR	NR
Staudacher *et al*(15)	NR	TN: 22 Non-TN: 40 P=0.008	NR	TN: 58 (62) Non-TN: 26 (51)	TN: 18 (19) Non-TN: 9 (18)	TN: 22 (24) Non-TN: 16 (33)
Koshy *et al*(12)	TN: 5.3 Non-TN: 1.7 P=0.058	TN: 47.8 Non-TN: 66.8 P=0.25	TN: 10.8 Non-TN: 4.3 P=0.10	TN: 7 (41) Non-TN: 3 (15)	TN: 0 Non-TN: 0	NR
Liedtke *et al*(16)	TN: 6	NR	13.5	21 (52.5)	6 (15)	7 (17.5)

TN, triple-negative breast cancer; NR, not reported in the text; PFS, progression-free survival; OS, overall survival; PBCT, platinum - based chemotherapy.
